# Implementation of the sensory organization test with the CAREN system: a pilot study

**DOI:** 10.3389/fbioe.2025.1635514

**Published:** 2025-10-17

**Authors:** Paolo De Pasquale, Augusto Ielo, Cristiano De Marchis, Daniele Borzelli, Antonino Casile, Antonio Caronni, Stefano Scarano, Rocco Salvatore Calabrò, Luigi Tesio, Angelo Quartarone, Andrea d’Avella

**Affiliations:** ^1^ IRCCS Centro Neurolesi “Bonino-Pulejo”, Messina, Italy; ^2^ Engineering Department, University of Messina, Messina, Italy; ^3^ Department of Biomedical and Dental Sciences and Morphofunctional Imaging, University of Messina, Messina, Italy; ^4^ Laboratory of Neuromotor Physiology, IRCCS Fondazione Santa Lucia, Rome, Italy; ^5^ Department of Neurorehabilitation Sciences, IRCCS Istituto Auxologico Italiano, Milan, Italy; ^6^ Department of Biomedical Sciences for Heath, University of Milan, Milan, Italy; ^7^ Department of Biology, University of Rome Tor Vergata, Rome, Italy

**Keywords:** CAREN, sensory organization test, balance, equitest, rehabilitation

## Abstract

**Introduction:**

The Sensory Organization Test (SOT) is a clinical and instrumental tool designed to assess postural stability by measuring body sway during standing under different sensory feedback conditions. This study explores the implementation of the SOT using the Computer Assisted Rehabilitation Environment (CAREN) system, aiming to enhance balance assessment and extend the diagnostic applications available for CAREN.

**Methods:**

A software application (CAREN-SOT) was developed to implement the SOT using the CAREN, which features a six degrees of freedom motion platform, force sensors, a 3D motion capture system, and an immersive visual environment. Eight healthy participants (ages 23–40, four males) underwent the SOT across six conditions, using either optic motion capture or force plate inputs to estimate the sway of the body center of mass. A generalized linear mixed model was employed to analyze equilibrium scores (ESs) from both modalities, considering system’s latency and responsiveness.

**Results:**

CAREN-SOT implementation was possible using both input modalities. No statistically significant differences were found between the optoelectronic and force plate modalities in measuring postural stability across conditions. Comparison with normative data from the NeuroCom™ EquiTest™ system suggested equivalence in key SOT metrics, despite minor variations in ESs likely due to methodological differences and sample size.

**Discussion:**

By integrating advanced technological and customization capabilities, CAREN-SOT provides an immersive, controlled environment for postural stability assessment. While findings must be validated on a larger sample, they support CAREN-SOT’s utility in diagnostic and rehabilitative settings. Future research directions include expanding normative datasets and exploring mediolateral sway to increase our understanding of postural control mechanisms.

## 1 Introduction

### 1.1 Postural and balance control

Postural control is a crucial motor skill for most daily living tasks. It can be defined as achieving, maintaining or restoring balance during any activity (Macpherson and Horak, 2013; Horak et al., 1996; Massion, 1994). Another definition, not applied here, relates “posture” to the “anticipatory” unconscious muscular “adjustments” coupled with voluntary muscular actions (anticipatory postural adjustments, APAs). In humans, maintaining balance during bipedal stance can be seen as a primary result of postural control, and APAs as one of its key components (Bouisset and Do, 2008). During stance, this is achieved by keeping the horizontal projection of the body’s center of mass (CoM) within its base of support (BoS) (Shumway-Cook and Woollacott, 1995; Winter 1995). It is widely acknowledged that sensory information from the visual, proprioceptive and vestibular systems contribute to postural control (Peterka, 2002). The visual system contributes to postural stability by providing information about the surrounding environment (Macpherson and Horak, 2013). Especially when visual feedback is reduced or absent, proprioceptive information from the foot/ankle becomes essential for identifying body sway for balance control (Goble et al., 2011). The vestibular system plays a role in maintaining balance by controlling the head’s and eyes’ movements and position (Macpherson and Horak, 2013; Tesio et al., 2013) and by triggering anti-gravity reactions along the body system (Fitzpatrick and McCloskey, 1994). Achieving and maintaining a stable upright stance is relevant to individuals of all age groups as it is essential for improving physical function throughout the lifespan (Van Humbeeck et al., 2023). Deficits in postural control (e.g., in older adults) have dramatic impact on the ability to perform daily activities safely and effectively. Furthermore, maintaining and enhancing postural stability is crucial for reducing the risk of falls, a major concern in our current ageing population. Therefore, assessing balance becomes essential in identifying individuals at risk and to timely implement preventive strategies to mitigate these risks and improve their overall quality of life (Alexander, 1994).

### 1.2 Testing postural control while standing

Various static and dynamic tests have been introduced to assess balance. The CoM moves with the BoS remaining stationary in static balance tests, and the BoS moves in dynamic tests. In either condition, the task can be static or dynamic (i.e., requiring the participant to remain steady or actively move). In static tasks, the paticipants’s goal is to keep the projection of the CoM within the BoS or the maximum stability limit, defined as the largest angle the CoM can sway without making the subject lose balance (e.g., taking a step, grasping a handle or falling) (Woollacott and Tang, 1997). Examples of static balance tests (i.e., with static BoS) during standing include the Romberg Test (Hong et al., 2015), functional Reach Test (Duncan et al., 1990), and BESS (Balance Error Scoring System) (Bell et al., 2011). Tests such as Tinetti’s POMA (Performance-Oriented Mobility Assessment) (Black and Nashner, 1983), the BBS (Berg Balance Scale) (Trueblood et al., 2018) and the mini BESTest (Vanicek et al., 2013) assess balance both statically and dynamically, providing a more comprehensive evaluation.

### 1.3 Computerized dynamic posturography: the NeuroCom EquiTest system

The above-described tests mostly rely on subjective, observation-based assessments. On the contrary, Computerized Dynamic Posturography (CDP) offers an objective quantification of balance control by isolating sensory contributions to postural stability (visual, proprioceptive and vestibular). CDP was designed and clinically studied by Black and Nashner (Black and Nashner, 1983), and commercialized in 1986 as EquiTest™ by NeuroCom™ (Int. Inc., Clackamas, OR, United States). The CDP test battery is currently considered the benchmark for static and dynamic balance testing during stance (Trueblood et al., 2018; Vanicek et al., 2013). In particular, the Sensory Organization Test (SOT), a subtest of the CDP, offers greater sensitivity and objectivity in evaluating postural stability compared to alternative assessment methods (Hong et al., 2015). The SOT determines balance by comparing postural sway, given in degrees of oscillations of the line connecting the CoM to the center of pressure (CoP) within the base of support, with the theoretical limit of stability (LoS) of 12.5° (of which, 4.5° backwards) in the anteroposterior plane (Perucca et al., 2021). The closer a person’s sway approaches this limit value, the higher the chance of a step being required or experiencing a fall.

A key component of the SOT is the estimation of the participant’s CoM, the location of which has been determined using various approaches over the years. These approaches fall into two primary categories: kinematic methods and methods based on the measurement of ground reaction forces. Two simple, yet effective, kinematic methods for measuring the CoM include the sacral marker displacement method, in which the sacrum bone is assumed to be coincident with the body’s CoM, and the more accurate reconstructed pelvis method. The latter is well approximated during an upright stance by calculating the spatial center of the pelvis segment, often identified through the left and right anterior superior iliac spines (LASIS and RASIS), and the sacrum (Saini et al., 1998). Methods that utilize force measurement typically employ a force platform. This platform records ground reaction forces (GRF) and uses them to calculate the location of the CoP, which only approximates the location of the CoM. The EquiTest system, which is equipped with a force plate, measures the CoP displacement and uses it to estimate the sway of the CoM to compute an Equilibrium Score (ES) and a Postural Stability Index (PSI) (Chaudhry et al., 2004; Chaudhry et al., 2011).

### 1.4 The CAREN system

Balance assessment is a critical diagnostic tool in clinical settings for evaluating fall risk and identifying underlying causes of balance disorders (Mancini and Horak, 2010). Notably, clinicians in rehabilitation settings often use a combination of clinical and instrumental balance assessments to create personalized rehabilitation programs (Scarano et al., 2022). To this end, the Computer Assisted Rehabilitation Environment (CAREN) system (Motek Medical B.V., Amsterdam, the Netherlands) offers promising opportunities for advancements in both diagnostics and rehabilitation practices (Formica et al., 2023; Kalron et al., 2016; Sessoms et al., 2015). Indeed, due to its extensive customization capabilities, the CAREN can be programmed to provide several balance assessments. In particular, it allows both kinematic and force plate methods to estimate the CoM displacement. The CAREN system available at the IRCCS Centro Neurolesi Bonino Pulejo in Messina (Italy) consists of a motion platform with six degrees of freedom (DOF) and a built-in dual-belt treadmill that can measure participants’ vertical, antero-posterior and lateral GRF in dynamic conditions. It is also equipped with a 180° screen surrounding the system and a motion capture system enabling realistic environmental interaction and precise movement data recording.

### 1.5 Concurrent validation of CAREN and EquiTest-derived SOT

This work aimed to assess the feasibility and potential advantages of implementing the SOT in the CAREN system (CAREN-SOT). Our final goal is to expand the scope of the SOT by using the CAREN’s advanced capabilities to generate an engaging and controlled virtual environment. In this report, we first describe the design, development and methodological details of the CAREN-SOT. We then present the results of the CAREN-SOT on a small sample of healthy subjects and compare them with normative data available in the literature to provide a pilot assessment of its feasibility.

## 2 Materials and methods

### 2.1 Sensory organization test

**FIGURE 1:**
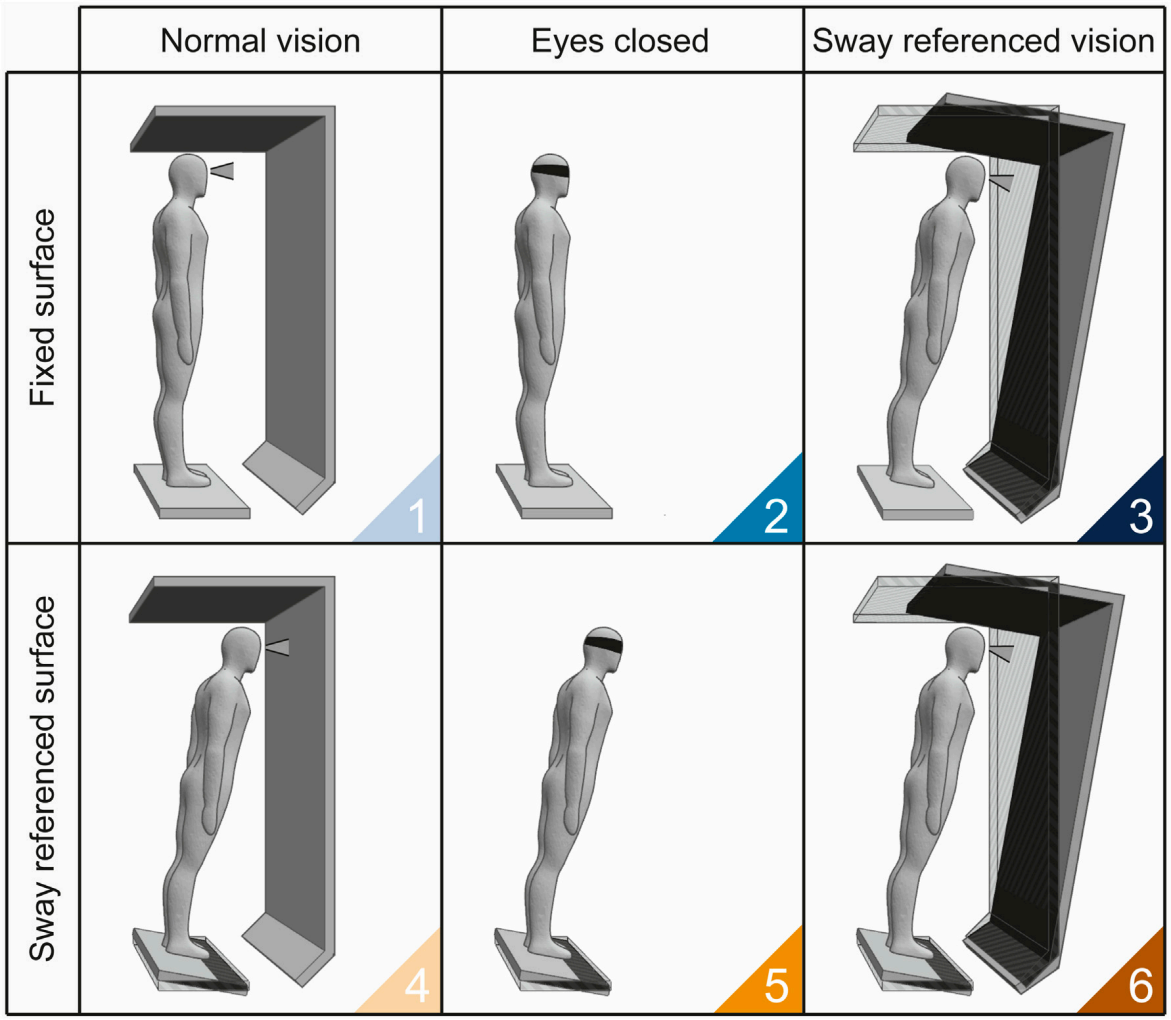
SOT conditions. 1) Normal vision - Fixed surface: this condition allows to test the ability to balance with all sensory systems providing reliable information and serves as a baseline for comparison; 2) Eyes closed - Fixed surface: evaluates the ability to balance without visual input, relying on vestibular and somatosensory information (so-called Romberg test); 3) Sway referenced vision - Fixed surface: as the participant balances with misleading visual information (i.e., moving visual environment “sway-tuned” with the CoM). This conditions is used to test reliance on vestibular and somatosensory inputs over conflicting visual cues; 4) Normal vision - Sway referenced surface: assesses the ability to balance with reliable visual and vestibular inputs but with misleading somatosensory information (i.e., moving platform); 5) Eyes closed - Sway referenced surface: tests the ability to maintain balance with accurate vestibular but compromised somatosensory feedback, in the absence of visual cues; 6) Sway referenced vison - Sway referenced surface: evaluates the vestibular system’s ability to override both unreliable somatosensory and visual inputs. SOT conditions are color-coded according to the sway reference (conditions 1-3, blue gradient; conditions 4-6, orange gradient).

The SOT measures the contributions of the visual, proprioceptive and vestibular systems in maintaining balance by computing an ES. The ES represents the collective efficiency of these systems in sustaining a steady upright position, measured in terms of anteroposterior oscillations of the CoM (Chaudhry et al., 2004). The EquiTest by NeuroCom Inc., consisting of a support surface or platform and a visual surround, is one of the earliest devices to perform the SOT, and still represents a gold standard. A SOT is typically carried out in six conditions (Figure 1): in conditions 1, 2, and 3 the platform is fixed and in conditions 4, 5, and 6 the platform moves; in conditions 3 and 6, the visual surround moves. In conditions 2 and 5, participants are instructed to close their eyes. The platform’s motion matches the participant’s body sway. For instance, if the subject leans forward, the platform tilts forward as well, minimizing the variation in proprioceptive feedback caused by the ankle movement. The platform adjustment is known as the “sway-referenced” motion. In conditions where the visual environment moves, it is adjusted in response to the subject’s oscillations to limit the ability to exploit visual information to detect individual’s deviations from the vertical axis. In conditions 2 and 5, where visual input is eliminated, the vestibular and the proprioceptive system (condition 2) and the vestibular system alone (condition 5) become more critical for maintaining balance due to missing visual information. It can be said that the SOT is a form of stress test on sensory systems, called to compensate for the systems that are absent (eye-closed condition) or unreliable (sway-referenced conditions). The same holds for condition 6, which also requires the capacity to suppress the tendency to privilege visual information (“visual preference”). Correspondingly, the balance is worse in condition 6 than in condition 5.

In this study the ES was calculated for each trial and condition according to Equation 1 (Chaudhry et al., 2011; NeuroCom International I, 2004):

$$ES=100\times \frac{tLOS-\left[\theta_{\max}-\theta_{\min}\right]}{tLOS}$$ (1)

where tLOS is the theoretical limit of stability in degrees in the sagittal plane for normal stance, $\theta_{\max}$ is the maximum sway angle in degrees during a trial, $\theta_{\min}$ is the minimum sway angle in degrees during the same trial. θ can assume positive values in case of anterior sway, and negative values in case of posterior sway.

In this study, as in the EquiTest system, tLOS was set to a value of 12.5° Chaudhry et al., 2011; NeuroCom International I, 2004). As the ES is a percentage indicating what fraction of the angle corresponding to theoretical limit of stability remained after subtracting the maximum observed angular oscillation $\left(\theta_{max}-\theta_{min}\right)$ , the higher the ES value, the lower the observed oscillation (Tesio et al., 2013).

A composite ES (CES) was calculated as a weighted average of the ES from the six conditions of the SOT of a participant as described in Equation 2 (Chaudhry et al., 2011; NeuroCom International I, 2004):

$$CES=\frac{ES_{1}+ES_{2}+3\times \left[ES_{3}+ES_{4}+ES_{5}+ES_{6}\right]}{14}$$ (2)

where $ES_{n}$ is the average of the ES in all the trials performed in condition n . The factor 3 multiplying ES in conditions 3-6 was introduced to increase the weight in the CES for the four most difficult conditions.

Four additional ratios were calculated for assessing standing balance and used in conjunction with the ES to identify impairments of individual sensory systems. The Somatosensory Index (SOM) quantifies maintaining balance without visual information. The Visual Index (VIS) measures the proficiency in suppressing proprioceptive information, thus relying primarily on vision. The Vestibular Index (VEST) evaluates the ability to sustain balance using solely vestibular information. Finally, the Visual Preference Index (PREF) measures the ability to suppress visual information when both proprioception and vision are unreliable. These ratios are calculated as described in Equations 3–6:

$$SOM=100\times \frac{ES_{2}}{ES_{1}}$$ (3)

$$VIS=100\times \frac{ES_{4}}{ES_{1}}$$ (4)

$$VEST=100\times \frac{ES_{5}}{ES_{1}}$$ (5)

$$PREF=100\times \frac{ES_{3}+ES_{6}}{ES_{2}+ES_{5}}$$ (6)

where $ES_{n}$ is the average of the ES in all trials belonging to condition n .

The manufacturer provides normative data for age groups from 20 to 79 (NeuroCom International I, 2004). More recently, normative values for subjects from 80 to 89 have been published (Perucca et al., 2021).

### 2.2 Implementation of SOT with the CAREN system

#### 2.2.1 Hardware setup

The CAREN is a multisensory system that integrates multiple technologies to provide a controlled and immersive rehabilitation environment. The system consists of a six DOF motion platform (MS V2, Moog, East Aurora, New York) supporting two independent force-instrumented treadmills (Forcelink BV, Culemborg, the Netherlands). A system with 13 infrared optoelectronic cameras (Vantage, Vicon, Oxford, United Kingdom) allows to track movements over the whole platform, and three projectors allow the display of virtual reality scenarios on a 5 m diameter, 3 m height cylindrical projection screen (spanning an angle of 180°) placed in front of the participant. A fourth projector allows to display images directly on the treadmill. For example, it shows the subjects where to position their feet during a test. A Dolby 5.1 surround sound system increases the feeling of immersion by providing audio feedback. The environment also includes safety suspension harnesses and handles to grab in case of balance loss. The concept behind CAREN is to acquire participants’ data from one of the input systems (either optoelectronic or force plates), process them in real time, and provide output feedback that can be visual, motor (platform and treadmill movement), auditory, or a combination of multiple outputs.

**FIGURE 2:**
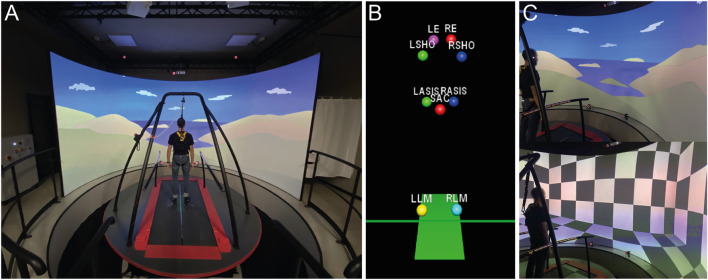
Implementation of SOT in CAREN. **(A)** Experimental setup; **(B)** Markers setup; **(C)** Visual scenes.

Here, we present an implementation of the SOT in the CAREN system (CAREN-SOT) (Figure 2A). In the NeuroCom EquiTest system, the rotation of the force plate and visual surround is controlled by two independent direct current servomotors, with a maximum platform pitch velocity of 50°/s and a maximum visual surround pitch velocity of 15°/s, the latter defining the system’s operational limits in terms of rotation velocity for the execution of the SOT. The maximum pitch velocity of the CAREN motion base is stated as 50°/s, while the visual feedback does not depend on mechanical properties but only on digital processing. The estimation of the CoM in the CAREN system was performed in separate trials, using either the kinematic (“marker input”) method or the kinetic (“force input”) method.

A set of nine markers was used for the experimental setup: LLM and RLM (left and right lateral malleolus of the ankle) were used to calibrate the pivot of the roto-translation; LASIS, RASIS and SAC (sacrum) were used to measure the CoM; LSHO and RSHO (left and right shoulder) were used to establish the positioning of the feet, providing a stable base of support; LE and RE (left and right eye) placed at the height of the eyes were used to adjust the participant’s point of view in the virtual environment (Figure 2B). The sway-referenced vision was implemented in the virtual environment by moving the visual scene on the screen. Two visual scenes were implemented: a virtual room with geometric patterns (i.e., checkerboard) and a naturalistic landscape (Figure 2C).

#### 2.2.2 Software and algorithms

The CAREN-SOT software was developed using the D-Flow suite (Motek Medical B.V), a visual programming tool for the development of interactive and immersive applications. It allows the creation of advanced applications by connecting visual modules to control hardware devices and virtual environments.

To implement the SOT’s sway-referenced conditions (vision and surface), we used two different strategies to estimate the participant’s oscillation angle (θ) based on the two input modalities (marker and force).

The angle (θ) was estimated using the inverted pendulum model through the variation (Δ) of the CoM as shown in Equations 7, 8:

$$\left\{\begin{array}{c} \Delta {\text{CoP}}_{z}={\text{CoP}}_{z}-{\text{CoP}}_{z,\text{init}}\;\;\;\;\left(\text{Marker input}\right)\;\\ \Delta {\text{CoP}}_{z}={\text{CoP}}_{z}-{\text{CoP}}_{z,\text{init}}\;\;\;\;\left(\text{Force input}\right)\; \end{array}\right.$$ (7)

$$\theta =\text{asin}\left(\frac{\Delta {\text{CoP}}_{z}}{{\text{Pelvis}}_{y}}\right)$$ (8)

Where x, y and z are the mediolateral, vertical and anterior-posterior axes, respectively. CoPz and CoPz are the projection of the reconstructed pelvis markers and the CoP displacement on the z-axis, while CoPz, init and CoPz, init are the reconstructed pelvis projection and the initial CoP displacement on the z-axis acquired during the calibration phase, respectively. ${Pelvis}_{y}$ is the mean height of the three pelvis markers evaluated during the calibration phase.

**FIGURE 3:**
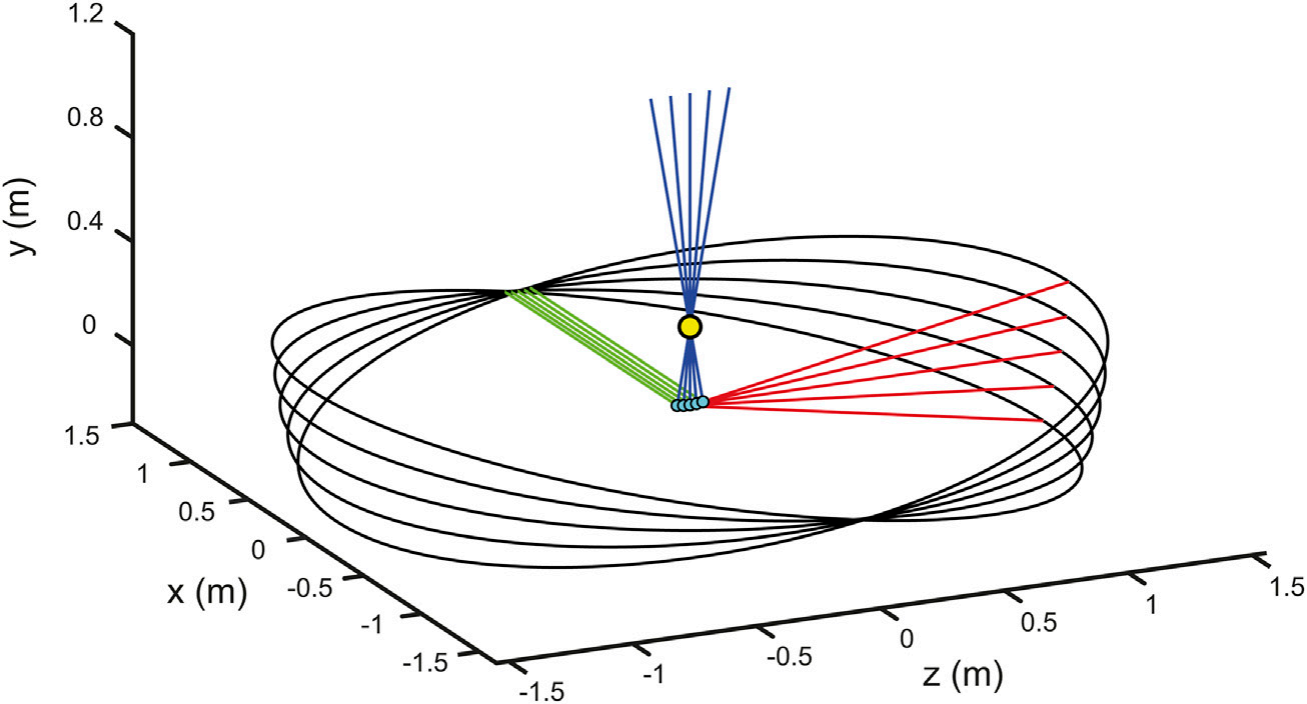
Platform roto-translation simulation. The figure shows a simulation of the platform movement around the ankles with different values of imposed angles of rotation (−10, 5, 0, 5, 10 degrees). The yellow marker indicates the position of the mean on x-axes of both ankles, the cyan markers indicate the projection of the ankle mean on the x-z platform plane. The green, blue and red lines indicate the platform’s Cartesian axes (x, y and z), respectively, in relation to the roto-translations.

Antero-posterior oscillations, estimated from the CoM or CoP variation depending on the control strategy, were transformed into translation and rotation movements of the platform and the visual environment (Figure 3), with the rotation axis passing through the subjects’ ankles. The motion calculation is shown in Equation 9:

$$\left\{\begin{array}{c} \Delta x=0\;\\ \Delta y={\text{CoP}}_{z}\times \sin\left(\theta \right)+{\text{Ankle}}_{y}\times \left(1-\cos\left(\theta \right)\right)\;\\ \Delta z={\text{CoP}}_{z}\times \left(1-\cos\left(\theta \right)\right)-{\text{Ankle}}_{y}\times \sin\left(\theta \right)\;\\ \Delta {\text{Rot}}_{x}=\theta \;\\ \Delta {\text{Rot}}_{y}=0\;\\ \Delta {\text{Rot}}_{z}=0\; \end{array}\right.$$ (9)

Where ${Ankle}_{y}$ is the mean value of LLM and RLM markers on the y-axis, $\Delta {\text{Rot}}_{x}$ is the rotation variation on the mediolateral axis. Δy and Δz are the position variations for the vertical and anterior-posterior axes, respectively.

### 2.2.3 Filtering and safety measures

Raw data acquired from the force plates used to control the motion of the platform and the visual scene were pre-processed by means of a second-order Butterworth low-pass filter to remove intrinsic measurement noise of the force sensors. The filter cutoff frequency $\left(f_{cut}\right)$ was estimated from preliminary tests as the maximum value allowing smooth platform movements (0.9 Hz). For data analysis purposes, the raw data recorded from the force plates were processed using a second-order Butterworth low-pass filter $f_{cut}$ = 15 Hz) to remove any artefacts (Stins et al., 2011). Data from the optical motion capture system were not pre- or post-processed. We neglected task-dependent soft-tissue artefacts, due to the small scale of the movements required to perform the test (Leardini et al., 2005).

In the NeuroCom EquiTest, the rotation of the force plate features a range of ± 10°, while the CAREN motion base has a rotation range of ± 19°. Thus, an adjustable rotation angle limit was introduced in our implementation of the CAREN-SOT for safety reasons. A check was performed on the angle θ (Equation 8) used to control the pitch in our CAREN-SOT system. Specifically, the pitch was restricted to vary in a given range (from ± 1° to ± 15°, set by default ± 10°). If the estimated angle θ was outside of that range, the system retained the previously estimated angle until a value within the allowed range was recalculated. We also implemented a further force threshold safety control whereby our software sets the minimum force required to safely perform the test for each force plate, detecting if the participant takes a step or detaches a foot from the platform. During the SOT execution in EquiTest, the operator manually interrupts the trial, whether the subject takes a step or touches the visual surround panel for support within 20 s, marking it as a fall or discarding it and repeating the process. In our CAREN-SOT implementation, these events, i.e., when the subject takes a step or lifts a foot, were automatically detected. Moreover, two custom capacitive touch sensors mounted in the platform handles are used to detect if the subject touches them and to automatically interrupt the trial. In such cases, an ES of 0 was assigned to the trial.

#### 2.2.4 Subject placement

The SOT guidelines suggest setting the width of the BoS according to the subjects’ height. Three fixed distances are printed on the force plates: Short = 20 cm, Medium = 25 cm, Tall = 30 cm, corresponding to 76–140 cm, 141–165 cm and 166–203 cm heights, respectively. Thus, lateral calcanei are positioned according to these parameters. In CAREN-SOT the base of support width is adjusted according to the individual subject’s morphometry. A rectangle with variable width is projected onto the platform before the start of the trial. The rectangle width is equal to the distance between shoulders (LSHO and RSHO markers) recorded during the initial calibration; the subjects’ lateral calcaneus must be aligned to the sides of the rectangle. Furthermore, a horizontal line passing through the center of the platform is projected, and the participants are required to align the LLM and RLM markers to the line.

#### 2.2.5 Video and platform delay

Depending on the selected input modality, the system responds with an output, which is the movement of the visual scene in the SOT conditions 3 and 6 (sway-referenced vision) or the movement of the platform in conditions 4, 5, and 6 (sway-referenced surface). In condition 6, both surface and visual scene are sway-tuned. The time difference between the estimation of the oscillation angle (θ) and the system movement response was measured to quantify the latency of the control system and assess its performance. To this end, we conducted six trials on 3 different subjects. The mean delay between input and output was calculated from the 3 repetitions (20 s each) of conditions 4 and 5 of the SOT. Each subject randomly underwent the test using control inputs (marker and force).

We used cross-correlation (xcorr, MATLAB R2022a, Natick, Massachusetts) to compare the θ calculated from marker and force inputs with the system response in rotation angles.

Movements of the visual scene are implemented in software, and they do not thus exhibit the latency that is instead exhibited by the mechanical actuators used to move the platform. To ensure consistency between visual feedback and physical motion of the platform we thus imposed a delay on the rotation of the visual scene that matched the measured mechanical delay of the actuators. To this end, we first estimated the latency of the platform’s response, and we then delayed presentation of the visual scene by the same amount.

The platform’s response latency generates a spatial error estimated for each condition as the mean of the absolute value of the difference frame by frame between inputs (θ) and outputs (platform rotation angle).

#### 2.2.6 Graphical user interface-GUI description

**FIGURE 4:**
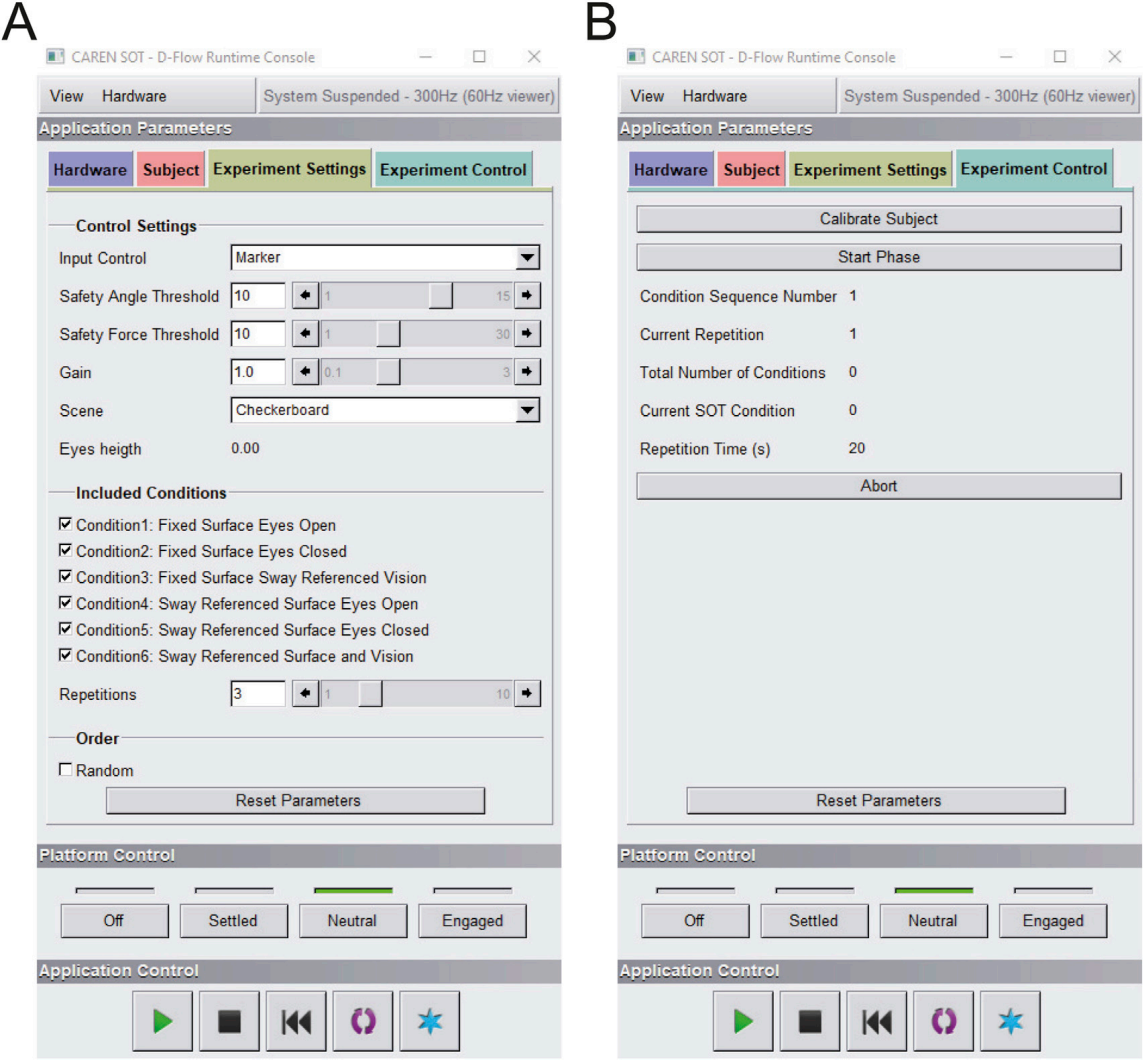
CAREN-SOT GUI (graphical user interface). **(A)** Experiment Settings tab: Interface for configuring experiment settings, including control parameters and conditions to be executed; **(B)** Experiment Control tab: Interface for executing the trials and monitoring the progress of the experiment.

We implemented, in the D-Flow suite, a graphical user interface (GUI) that allow the operator to control the experiments. In addition to a default Subject panel, where participant data can be entered (i.e., identification code, gender, age), our CAREN-SOT GUI includes two custom panels that allow for the configuration of the experimental parameters and the real-time control of the experiment, respectively. Within the “Experimental Settings” tab (Figure 4A), it is possible to set the “Input Control” type, which enables the selection of the desired input modality (i.e., marker or force). Two safety values can be specified—the first one, named “Safety Angle Threshold”, sets the maximum pitch angle of the platform (in degrees). The second, “Safety Force Threshold,” sets the minimum force threshold for each platform (N), indicating when the participant lifts a foot from the platform. Both safety values prevent conditions that could lead to a loss of stability by terminating the current trial. The “Gain” parameter amplifies or reduces the ratio between the commanded (visual and/or platform) angle and the estimated, sway-referenced oscillation angle. The system can amplify the angle up to three times or reduce it to a tenth, with a default value set to 1. The “Scene” parameter allows the selection of the type of visual scene between the two developed options (checkerboard or landscape). The “Eyes height” label reports the subject’s eye height, which is recorded through LE and RE markers. The “Included Phases” section allows selecting the SOT conditions to be performed, while the “Repetitions” parameter defines the number of sequential repetitions of trials for each condition (by default, 3). Through the “Order” section, the operator can choose to perform them in ascending order or randomly.

Once the experimental parameters are set, the experiment proceeds with the execution, starting with the 5-s recording of the average position of hip, ankle, and eye-level markers using the “Calibrate Subject” button. These are stored as reference values with respect to which the oscillation angle, system rotation, and camera viewpoint are calculated. The “Experimental Control” tab (Figure 4B) includes the features used for the real-time execution and monitoring of the test. The “Start Phase” button allows to initiate the next phase in the predetermined order (ascending or random) and must be manually clicked by the operator. A series of indicators shows the current phase and acquisition time. If necessary, a button allows you to abort the current trial and to repeat it. Optionally, using the “Reset Parameters” button, parameters can be set to default values.

#### 2.2.7 Experimental test

Eight healthy subjects (four females) were enrolled in a pilot study (age range 23–40 years, mean value and standard deviation (SD) age: 32 ± 6 years; height: 168 ± 6 cm, mass: 60 ± 7 kg). Before the testing session, all participants were instructed to refrain from alcohol consumption for 12 h and coffee and cigarettes for 2 h (Tesio et al., 2013). All experiments were conducted according to the ethical policies and procedures approved by the local ethics committee (IRCCS-ME-23/2022). All participants gave their written informed consent.

Anatomical landmarks were identified using palpatory anatomy techniques, and reflective markers were attached to the skin with double-sided adhesive tape to ensure stability and minimize artifacts. All marker placements were performed by a single trained physiotherapist with expertise in palpatory anatomy, to ensure consistency across participants and avoid inter-rater variability. All participants familiarized themselves with the CAREN-SOT prior to performing the test to improve its reliability (Summers et al., 2022) and minimize the subject’s movement unrelated to postural control in response to unexpected platform movements.

The participants performed the six SOT conditions (each trial lasting 20 s) in ascending order from 1 to 6. Each condition was repeated for three consecutive trials. At each trial an ES ranging from 0 to 100 was computed, with ES equal to 0 indicating a “fall” (a stepping reaction, hands touching the surround, or falling and being supported by the safety jacket) and an ES equal 100 indicating complete stability throughout the trial (never attained in practice).

Each participant performed the six SOT conditions with both input control modalities (marker and force). Participants first completed all conditions with one modality and, after a 10 min break, repeated them with the other. The order of modalities was assigned to participants in an alternating sequence, while ensuring gender balance. At the beginning of the test, all subjects were placed in the same initial position as described in the “Subject placement” section. Participants wore a safety harness while performing the CAREN-SOT.

#### 2.2.8 Statistical analysis

The relationship between the ES and the experimental factors was investigated using a generalized linear mixed model (GLMM) that accounts for interindividual variability by including the participant as a random effect. GLMM was chosen because of the non-normal distribution of some of the data. The gamma distribution was selected for the response variable due to its suitability for modelling positive continuous data with skewed distributions. A log link function (MATLAB function file) was used to linearize the relationship between the dependent variable and model predictors. The SOT conditions (Macpherson and Horak, 2013; Horak et al., 1996; Massion, 1994; Bouisset and Do, 2008; Shumway-Cook and Woollacott, 1995; Winter 1995), i.e., Conditions (C) and Input (I), were treated as fixed effect factors with categorical (dummy) variables. Data were thus fitted with the model described in Equation 10:

$$Y=g\left(u_{0}+\alpha_{0}C+\beta_{0}I+\lambda_{0}CI+\epsilon \right)$$ (10)

Where $u_{0}$ represents the individual intercept and accounts for interindividual differences. The coefficients $\alpha_{0}$ , $\beta_{0}$ and $\epsilon_{0}$ represent fixed effects, thus the modulation of the response variable by the main factors C and I and their interaction. In the equation, g represents the link function. The estimation of model parameters was based on the maximum likelihood using Laplace approximation. Dummy variables for fixed effects C and I were defined for the corresponding conditions with the highest mean ES (SOT condition 1) in the marker input modality. Post-hoc comparisons were performed by assessing the p-values of the regression coefficients for the dummy variables and their interactions.

CES percentage differences between normative data for healthy adults (age 20–59 years) provided by NeuroCom (NeuroCom International I, 2004) and for CAREN-SOT’s different control modalities (marker and force) were calculated to evaluate the reliability of the implemented methods.

Consistency between the marker input scores and the force-plate input scores for each participant was assessed calculating the Intraclass Correlation Coefficient (ICC) (McGraw and Wong, 1996). A two-way random effects model with single measurements and a consistency definition [ICC(2,1) for consistency] was employed. Furthermore, a single ICC was computed across all equilibrium scores (ES) obtained from both modalities and all subjects. Based on the 95% confidence interval of the ICC estimate, values below 0.5 indicate poor reliability, values between 0.5 and 0.75 indicate moderate reliability, values between 0.75 and 0.90 indicate good reliability, and values above 0.90 indicate excellent reliability (Koo and Li, 2016; Tesio, 2012).

## 3 Results

We implemented the SOT in the CAREN system, using two different input modalities: one based on optical markers and the other on the force plates. In the following, we describe the results of the tests conducted to characterize the system’s movement performance and to determine its suitability for executing the SOT.

### 3.1 Performance delay

**FIGURE 5:**
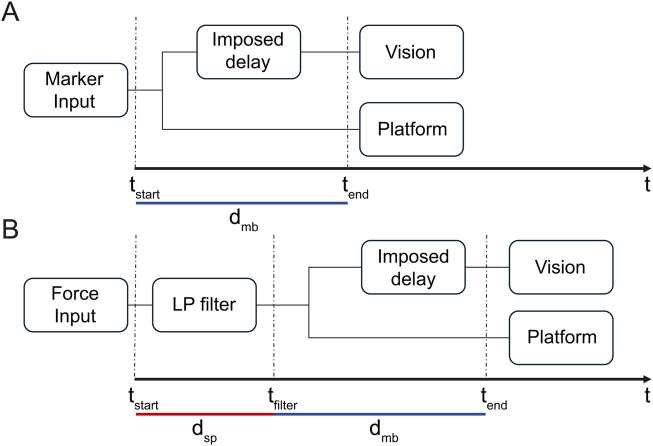
System timing diagram. The figure shows the timeline of responsibility for the CAREN-SOT system, from data acquisition to platform and vision outputs for marker input **(A)** and force input **(B)**.

The platform movement performance in response to the recorded oscillation angle was assessed by recording six trials from 3 participants and estimating the delay in platform response. The delay is influenced by both the physical characteristics of the platform ( $d_{mb}$ , platform delay), attributable to the actuators, and the signal processing ( $d_{sp}$ , signal processing delay). While the platform’s physical characteristics (mostly inertia) affect the delay with both input modalities, signal processing exclusively affects the force input control mode (Figure 5). To prevent unsafe and uncontrolled platform movements, the raw force input, characterized by intrinsic sensor measurement noise, had to be low-pass filtered before inputting it to the platform control system. For this reason, the force input modality exhibits a larger delay compared to the marker input modality that does not require filtering.

Figure 5 shows the system’s timing diagram from data acquisition to platform and visual output from marker input (A) and force input (B). The time between input $\left(t_{start}\right)$ and output $\left(t_{end}\right)$ is due only to delay $\left(d_{mb}\right)$ in case A. In contrast, in case B, it is due to both the delays $d_{mb}$ and $d_{sp}$ .

We found a total delay between input $\left(t_{start}\right)$ and output $\left(t_{end}\right)$ of 0.28 ± 0.05 s (mean value ± SD) for marker input (with a peak cross-correlation r = 0.97 ± 0.01), and 0.54 ± 0.04 s for force input (r = 0.57 ± 0.08). We also estimated the two separate delays due to the motion base and the signal processing ( $d_{mb}$ = 0.29 ± 0.04 and $d_{sp}$ = 0.20 ± 0.01 s, respectively) for the force input ( $r_{mb}$ = 0.96 ± 0.01, $r_{sp}$ = 0.60 ± 0.09). We computed a unique $d_{mb}$ for both input modalities ( $r_{mb}$ = 0.97 ± 0.01), averaging the two values ( $d_{mb}$ = 0.29 ± 0.04 s). Based on these results, we delayed the movement of the visual scene to compensate for the estimated delay.

We found an average angle error between subjects’ oscillations and the platform response of 0.19 ± 0.06° for the marker input and 0.27 ± 0.04° for the force input.

### 3.2 SOT scores

**FIGURE 6:**
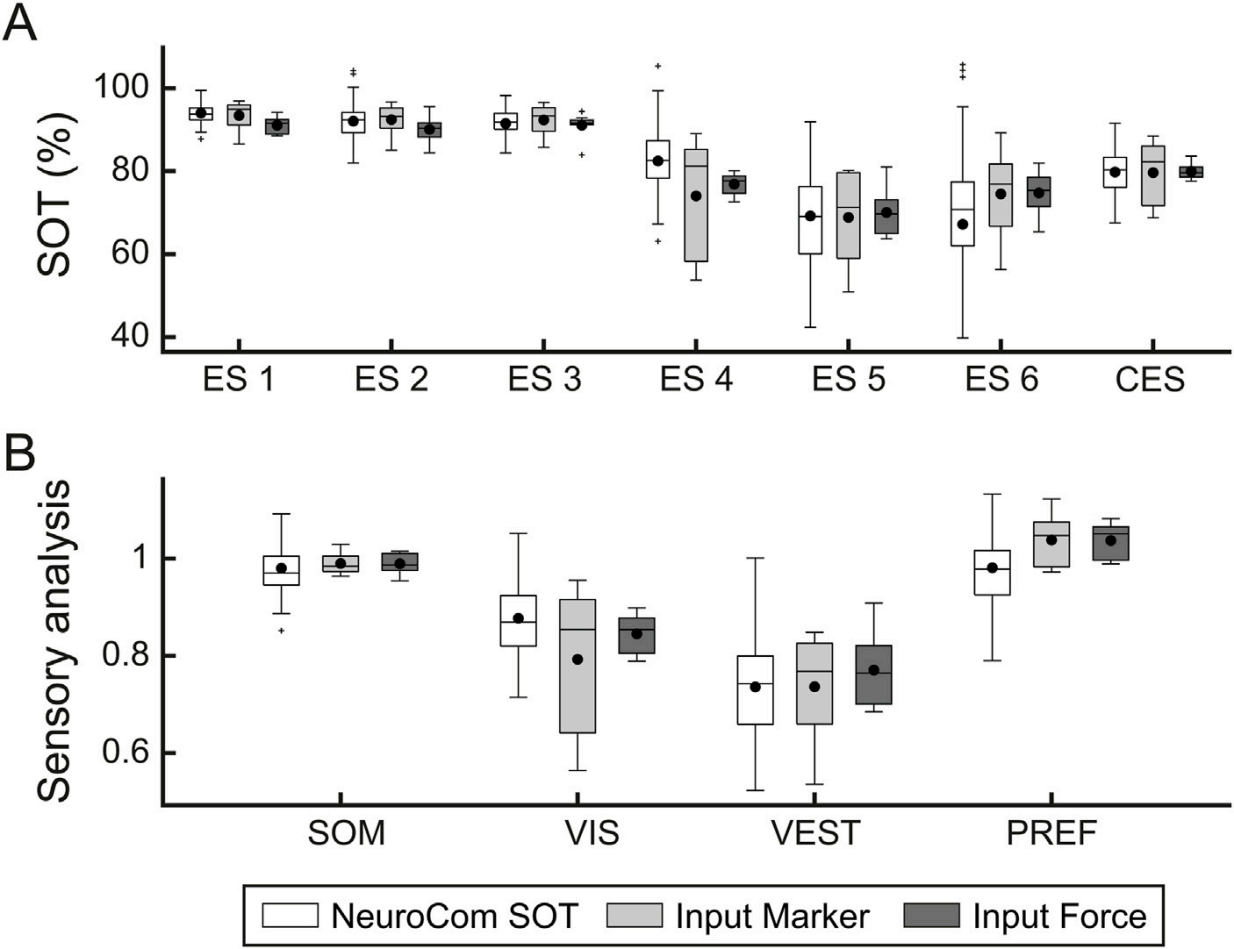
SOT and sensory analysis scores comparison between NeuroCom normative data and CAREN-SOT marker and force input controls results. The figure shows boxplots of the six SOT conditions scores **(A)** and the sensory analysis **(B)** for NeuroCom EquiTest (white), marker input (light grey) and force input (dark grey) data. Data shown in the “NeuroCom SOT” boxplots were generated, for illustrative purposes only, by creating a Gaussian distribution of 121 data points based on the normative mean and standard deviation values from the NeuroCom manual (age range 20–59 years) (NeuroCom International I, 2004).

The results for both input modalities are reported for SOT and sensory analysis performed on 8 healthy subjects. Figure 6 shows the six SOT conditions scores (A) and the sensory analysis (B) for NeuroCom EquiTest data (white boxplots) and both input controls modalities (marker, light grey boxplots; force, dark grey boxplots). The mean values for the ESs and CES evaluated over the 3 repetitions and the sensory analysis scores for all subjects are reported with black circular markers. The GLMM applied to CAREN-SOT scores of conditions 1-6 of the two inputs ( $R^{2}$ = 0.57) indicated a statistically significant effect of the condition (p < 0.001). There was no statistically significant effect of the input modality (p = 0.42) and no statistically significant Condition × Input interaction (p = 0.55), although there was higher variability in the ESs for the marker input (Figure 6A).

Indeed, the SOT conditions involving platform movement had a larger interquartile range (IQR) for marker input compared to force input. For instance, the IQR for condition 4 of the marker input ranges from 58 to 85, whereas the IQR for the force input ranges from 75 to 79. In the sensory analysis (Figure 6B), the difference in variability can be observed only in the VIS score, which depends on the $ES_{4}$ (Equation 4), where the IQR of the marker input ranges from 0.64 to 0.92, while the IQR for the force input ranges from 0.81 to 0.88.

Notably, the median values for both input modalities of each score of the sensory analysis are very close despite the observed variability. Mean CES results were compared between NeuroCom (mean ± SD = 80 ± 6%), marker input (mean ± SD = 80 ± 8%), and force input (mean ± SD = 80 ± 2%). We found a difference between estimated CES and NeuroCom normative data of 0.18% for marker input and 0.20% for force input.

**FIGURE 7:**
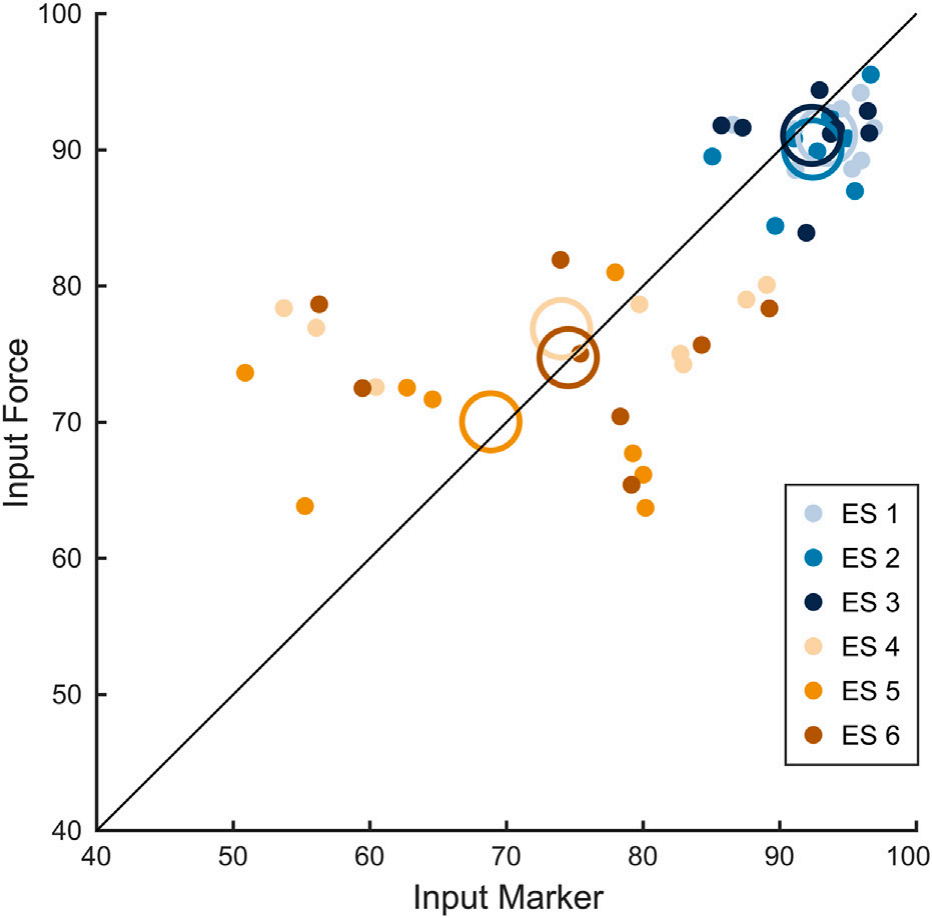
Equilibrium scores for marker and force input control modalities. Figure shows the scatter plot with 1:1 line (black) between marker and force inputs for each condition (different color-coded circular markers) for each subject averaged across repetitions. Empty color-coded circles show the mean value across all subjects for each condition.

Figure 7 shows the relationship between the scores computed according to the two input modalities across all the conditions, for each subject and condition (small filled colored circular markers), averaged across repetitions. Conditions with no platform movement (1-3, blue markers) showed a ratio between inputs close to 1, while the sway referenced surface conditions (4-6, orange markers) exhibited greater variability between inputs. However, this variability appeared random, as the mean across subject for each condition (large colored circular markers) were close to the 1:1 line. Moreover, it is worth noting that for the marker input, the ES under sway referenced surface conditions showed greater variability compared to force input scores. The Figure confirms that the two input modalities had on average, a linear relationship and that the variability depends on the subjects and/or, the subjects × condition interaction.

**FIGURE 8:**
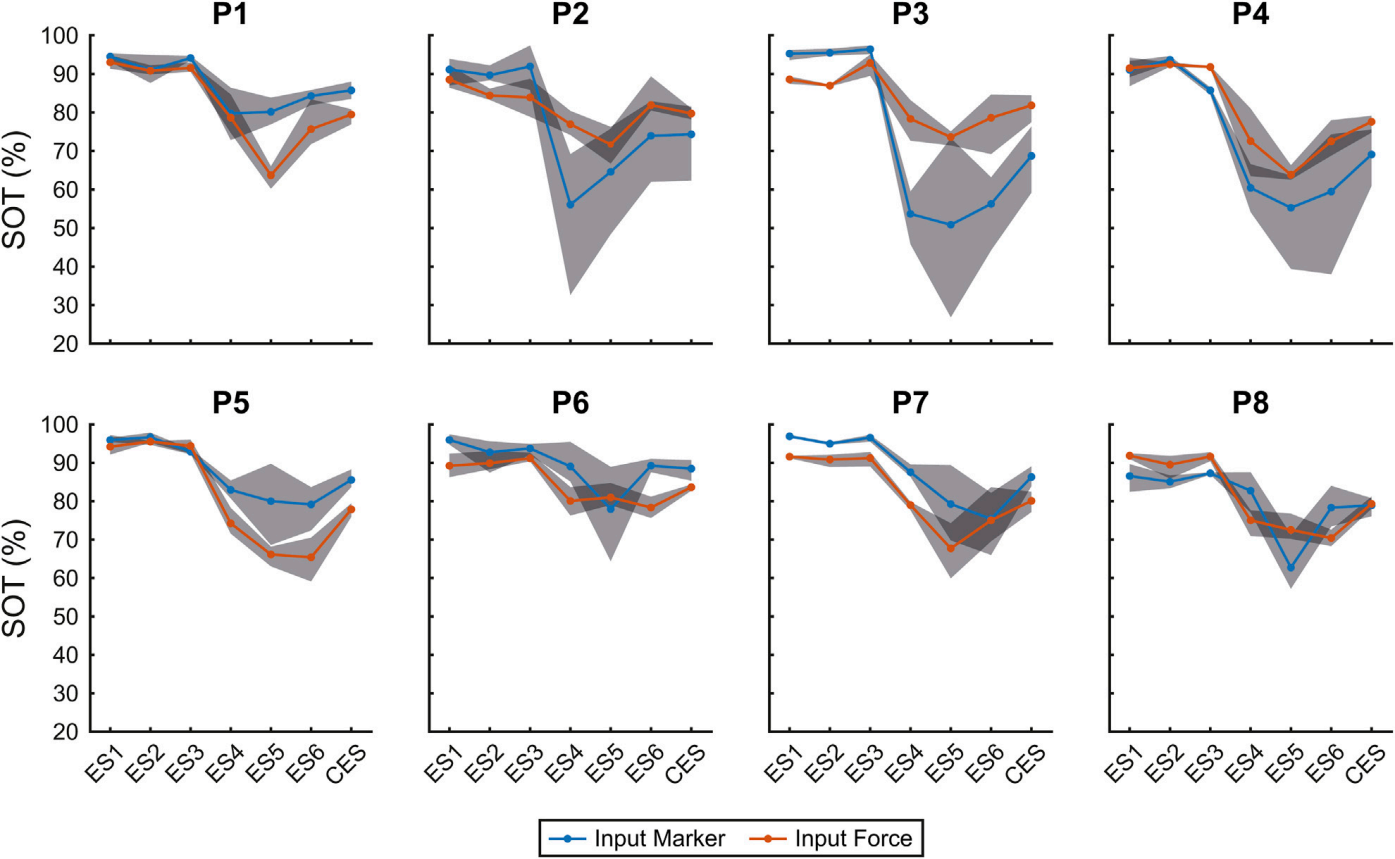
SOT scores for individual participants. The figure shows the SOT scores for each participant (P), with separate subplots illustrating the mean values and range (grey area) for both marker (blue) and force (red) inputs across the six SOT conditions and CES.

Figure 8 helps to assess the role of subjects and conditions in generating scores’ variability. The figure shows, for each participant in a separate panel, mean values and range (represented by the grey areas) for both marker (blue) and force (red) inputs of the SOT scores across the six conditions and the CES, illustrating the consistency pattern across conditions. The results of the ICC analysis revealed varying degrees of consistency across participants. For participants 2, 3, 6 and 8, the ICC values were 0.57, 0.55, 0.65 and 0.71, respectively, indicating moderate reliability between the two input methods. Participants 1 and 5 exhibited higher ICC values of 0.78 and 0.84, suggesting good agreement, while participants 4 and 7 demonstrated excellent consistency with ICC values of 0.93 and 0.92, respectively.

The overall ICC(2,1) computed across all subjects and SOT conditions was 0.64 (95% CI: [0.46, 0.77]), indicating a moderate level of agreement between the marker-based and force-based input modalities. In all cases, variability was higher for the moving platform conditions. Results suggest that these conditions contribute to the high score variability, independently of the subject.

## 4 Discussion

The results of this pilot study demonstrate the feasibility of implementing the SOT in the CAREN system using either marker-based optical tracking or force plates to estimate body sway. The development of the CAREN-based SOT involved a detailed estimation of the CoM oscillations based on individual participant morphology. We modeled the participants’ movements with greater precision by leveraging 3D positions of bony landmarks, such as the ankle, pelvis rotation axes, and eye height. In particular, we modeled the ankle motion on the sagittal plane as a roto-translation, not as a uniaxial rotation as in the EquiTest and other posturographic systems (NeuroCom International I, 2004). The CAREN thus allows a closer approximation of the complex motion of the foot on the leg. Additional features of the CAREN-based SOT are the customization of the BoS width and the field of view, making it adaptable for various participants, including those with specific physical or motor impairments.

We characterized the system latency, which is defined as the time lag between the CoM oscillation and the motion of the platform and the visual surround. In both input modalities (marker and force), latencies were introduced by the mechanical inertia of the platform and the filtering process of the force sensors’ readings. We found mean latencies of 0.54 s in the case of force input and 0.28 s in the case of marker input. These values, although within temporal ranges tolerated by multisensory integration and postural feedback (Peterka, 2002; Fister et al., 2016; Trivedi et al., 2010; Wa et al., 2014; Zerr et al., 2019), are higher than the ideal thresholds for real-time sway tuning. Such delays may therefore influence sensory reweighting and compensatory strategies in sway-referenced conditions, even if they do not compromise the overall validity of SOT performance. The CAREN platform’s greater inertia compared to the lighter EquiTest platform likely contributes to these delays. However, the latencies of the EquiTest system are not known, making it difficult to directly compare the responsiveness of the two systems.

We also estimated the average angle error between CoM oscillation and platform movements to determine how effectively the CAREN platform mitigates unintended ankle movements and preserves proprioceptive feedback. We found an angle error of 0.19 degrees for the marker input and 0.27 degrees for the force input. Although these deviations are small, accurate proprioceptive feedback is essential for stabilizing sway (Chiari et al., 2000; Horak et al., 2002), and even minimal errors may contribute to subtle perceptual or motor biases in challenging balance tasks, with potential implications for diagnostic accuracy. Unlike other assessment systems (Perucca et al., 2021; Leardini et al., 2005; Summers et al., 2022), we could estimate this error leveraging the advanced kinematic measures provided by the CAREN.

Despite these limitations, the results from the CES satisfactorily matched the normative data from the EquiTest system (Leardini et al., 2005), indicating that the differences in tuning did not significantly affect the average of the six ES and the CES. This finding speaks against the difference in variability in the effect of “tuning” between the two systems, which could be due to the different responsiveness of the two input modalities. Nevertheless, the ICC findings indicate that both input modalities generally provided reliable data for most participants. It is worth mentioning that a significant difference between the CAREN-SOT results and the NeuroCom normative data was observed in conditions 5 and 6, where $ES_{5}$ was lower than $ES_{6}$ in the CAREN-SOT compared to normative data. In contrast, normative NeuroCom data show lower scores in condition 6 compared to condition 5, a result attributed to the difficulty to counteract the tendency to privilege visual information. One possible explanation for this difference involves how the visual surrounds of the two systems respond to subject sway. In the EquiTest system, the visual surround consists of a mechanically driven panel with its own inertia and delay, potentially leading to different response characteristics compared to the CAREN-SOT. Furthermore, the delay imposed on the visual environment to match the inertia of the CAREN platform may have influenced how the visual environment was perceived or how rapidly participants could adjust to visual perturbations. Although this pilot study was not designed to directly test these mechanisms, future studies will specifically investigate the effect of manipulating visual delays within CAREN in order to clarify their impact on SOT outcomes.

Moreover, the small sample size of the present pilot study, together with the absence of an *a priori* power analysis, limits the statistical strength and generalizability of the results. This notwithstanding, the CAREN-SOT provided mean results matching the EquiTest-SOT norms, although a higher variance, presumably not systematic, was observed in some conditions. In addition, the ecological validity of the study is limited, as only healthy young adults were tested under controlled laboratory conditions. However, an important advantage of the CAREN system compared to traditional platforms such as the NeuroCom EquiTest is the integration of immersive VR, which allows the creation of complex and ecologically valid scenarios that more closely replicate real-life balance challenges. Future studies should therefore include broader age ranges and clinical populations, and exploit the VR component of CAREN to strengthen translational applicability in both assessment and rehabilitation contexts.

Despite the CAREN-SOT having proven to be a promising tool for the balance assessment, the methods used in our study are based on an approximation of the CoM position through the inverted pendulum model, as in the NeuroCom system. Moreover, both the two developed control modalities can suffer from inaccuracies due to their own technological constraints. For instance, optoelectronic methods such as sacral marker or reconstructed pelvis can be affected by marker placement inaccuracies and soft tissue artefacts (Saini et al., 1998; Leardini et al., 2005). Force-based models are limited by the simplified assumption of ankle-only control, difficulties in differentiating CoM from CoP under high sway frequencies, lack of standardization in foot posture, and sensitivity to anthropometric variations (Chen et al., 2021). Furthermore, they suffer from dependence on CoP filtering, inaccuracies with complex motions like hip strategy, and drift in force measurements (Sonobe and Inoue, 2023). Although our statistical analysis did not show significant differences between the SOT scores obtained using the two models, the shorter latency observed and the smaller angle error with marker control suggest it may be more effective for real-time applications, offering greater responsiveness to body oscillations and potentially improving the accuracy of the balance assessment. Further refinement of both models is necessary to improve the accuracy of CoM estimation, especially for dynamic and multi-planar balance assessments.

Additionally, the CAREN system’s greater complexity, cost, and space requirements compared to the EquiTest are offset by its flexibility and potential for customization, making it a powerful tool for clinical assessments and therapeutic applications, which the EquiTest system does not foresee (Tesio et al., 2013; Chang et al., 2004). The CAREN system, with 30 units worldwide (20 CAREN Extended and 10 CAREN High End), offers opportunities to tailor tests to patients with varying anatomical, motor, and cognitive impairments without the need for a dedicated SOT assessment system. Moreover, its application in therapeutic settings, due to its ability to provide immersive and interactive environments, could open new avenues for rehabilitation.

Over the years, various systems have been developed to assess postural control and allow the administration of the SOT. These systems vary in performance and cost (Trueblood et al., 2018; Wagner and Merfeld, 2023), but they are designed for specific purposes, making them less customizable than the CAREN, a general-purpose system. However, custom systems based on virtual reality headsets, which allow for the recording of kinematics (De Pasquale et al., 2024), have also been developed to perform virtual reality-based SOT, although they do not provide the sway-referenced surface condition (Wittstein et al., 2020).

The CAREN also allows lateral oscillations of the platforms, thus lending itself to expand the SOT approach to this plane (where balance, at least in walking, is more critical than in the sagittal plane) (Malloggi et al., 2021). The CAREN is also an advanced system for gait analysis (Bonanno et al., 2023). This offers the unique opportunity to compare balance during stance and walking on the same device in the same testing session. Measuring balance during walking is an emerging challenge in the literature and can widen clinical knowledge on balance deficits in the most various impairments (Fitzpatrick and McCloskey, 1994; Tesio et al., 2011; Tesio and Rota, 2019).

Future studies will be conducted to establish normative data on larger samples of healthy subjects. Further research should also include the assessment of mediolateral and combined anterior-posterior oscillations (Chang et al., 2004; Malloggi et al., 2021) to enrich our understanding of balance control mechanisms.

## 5 Conclusion

In conclusion, with its enhanced platform tuning, real-time control methods, and immersive VR the CAREN-SOT system provides a sophisticated and customizable tool for postural stability assessment. Key findings of this pilot study include the overall agreement between marker-based and force-based input modalities, the comparability of CAREN-SOT results with EquiTest normative data, and the added value of advanced kinematic modeling of the CoM and customizable testing conditions. Functionally, these strengths enable a more precise characterization of balance strategies in both static and sway-referenced conditions, while clinically they support tailored assessments and therapeutic applications for individuals with diverse motor or sensory impairments. At the same time, the technology’s applicability is currently bounded by latency differences between modalities, the relatively small sample size, the absence of *a priori* power analysis, and the limited ecological validity of testing only healthy adults under laboratory conditions. However, the CAREN system’s integration of immersive VR represents a unique opportunity to create ecologically valid and patient-specific scenarios, extending the test beyond what traditional systems can offer. Future studies should therefore expand normative datasets, include broader and clinical populations, and explore more complex and dynamic balance scenarios to fully harness the translational potential of this technology in both assessment and rehabilitation.

## Data Availability

The raw data supporting the conclusions of this article will be made available by the authors, without undue reservation.
